# Cancer patients with community-acquired pneumonia treated in intensive care have poorer outcomes associated with increased illness severity and septic shock at admission to intensive care: a retrospective cohort study

**DOI:** 10.15172/pneu.2015.6/645

**Published:** 2015-12-01

**Authors:** Ricardo J. P. José, Ali O. Mohammed, James J. P. Goldring, Rachel C. Chambers, Jeremy S. Brown, Banwari Agarwal

**Affiliations:** 110 Centre for Inflammation and Tissue Repair, Department of Thoracic Medicine, University College London Hospital, 5 University Street, London, WC1E6JF UK; 210 Department of Intensive Care Medicine, Royal Free Hospital, London, UK; 310 Department of Thoracic Medicine, Minia University, Elminia, Egypt; 410 Department of Respiratory Medicine, Royal Free Hospital, London, UK; 510 Centre for Inflammation and Tissue Repair, University College London, London, UK

**Keywords:** pneumonia, cancer, sepsis, ICU, outcomes

## Abstract

Patients with community-acquired pneumonia (CAP) and an underlying diagnosis of cancer have worse outcomes. However, the characteristics of cancer patients with CAP admitted to intensive care units (ICUs) are not well established. In a retrospective observational study, patients admitted to a London university hospital ICU between January 2006 and October 2011 with a primary diagnosis of CAP were included. Demographic, clinical, laboratory, and outcome data were collected from the ICU and hospital pathology databases. The analysis included 96 patients with CAP, 19 of whom had an existing diagnosis of cancer. Patients with cancer had a longer median time interval between hospital and ICU admission (1 vs 2 days, *p* = 0.049). On admission to ICU, there were no differences in white cell count, C-reactive protein, clotting, renal function, liver function, heart rate, temperature, systolic blood pressure or oxygenation index between patients with or without cancer. However, patients with cancer had significantly lower haemoglobin levels (median 8.6 vs 10.0 g/dl, *p* = 0.010) and lowest diastolic blood pressure (median 40 vs 50 mmHg, *p* = 0.026), and higher sodium levels (median 142 vs 139 mmol/l), *p* = 0.020), APACHE II (median 25 vs 20, *p* = 0.009), SAPS II (median 51 vs 43, *p* = 0.039) and SOFA (median 12 vs 9, *p* = 0.018) scores. There were no statistically significant differences in the proportion of patients receiving mechanical ventilation or renal support, the duration of mechanical ventilation or ICU or hospital length of stay. Patients with cancer were more likely to receive vasopressors (89.5% vs 63.6%, *p* = 0.030) and had increased ICU (68.4% vs 31.2%, *p* = 0.004) and hospital (78.9% vs 33.8%, *p* = 0.001) mortality. The limitations of this study are its relatively small sample size and those associated with the retrospective study design. In conclusion, cancer patients with CAP had an increased risk of death that was associated with increased illness severity and prevalence of septic shock at the time of ICU admission, suggesting there may be a delay in recognition for the need for intensive care support in these patients.

## 1. Introduction

Patients with cancer have immune dysfunction either related to the underlying malignancy or as of result of treatment and this puts them at increased risk of developing life-threatening infections, particularly pneumonia [1], Indeed, 50% of cases of septic shock in cancer patients are caused by bacterial lung infection [2]. Additionally, patients with community-acquired pneumonia (CAP) that have an underlying diagnosis of cancer have worse outcomes irrespective of absolute neutropenia or the prognosis associated with the underlying malignancy [3,4], The characteristics of cancer patients with CAP admitted to intensive care units (ICUs) may partially explain the discrepancies in outcomes between patients with and without cancer, but are not well established. In this observational study we have compared the characteristics and outcomes of CAP patients with and without cancer admitted to our ICU.

## 2. Methods

### 2.1 Study design and setting

The reporting of this observational study is compliant with the standards of the STROBE Statement. A retrospective review of prospectively, routinely collected data of consecutively admitted patients to the ICU at the Royal Free Hospital, London, with a primary diagnosis of CAP (with or without a diagnosis of cancer) was undertaken as part of a service evaluation. The dataset included the period from January 2006 and October 2011. The Royal Free Hospital Research and Development department waived the need for patient consent. Patient records were de-identified prior to analysis.

### 2.2 Study population and data collection

Data of all inpatients with a coding diagnosis of pneumonia (bacterial pneumonia, viral pneumonia, fungal or yeast pneumonia or pneumonia, no organism isolated) was retrieved from our ICU database, which is part of the national intensive care database, the ICNARC (Intensive Care National Audit & Research Centre), UK. The CAP diagnosis was validated by reviewing all cases in the dataset. We included those cases with typical clinical symptoms consistent with respiratory infection (dyspnoea, cough, sputum production, fever [temperature ≥38°C]) and evidence of new radiographic shadowing within 48 hours of admission to hospital that was not known to be due to other causes. Patients with a previous hospital admission within 14 days and those admitted from nursing homes were excluded. To address issues with information bias, case notes review was carried out by the authors (AM, RJ, BA) and the information cross-referenced with that in the ICU database and radiology database. All chest radiographs were reviewed by the requesting clinician and usually reported within 48 hours by a consultant radiologist. For this study, all chest radiographs were reviewed by one of the authors (AM, a senior respiratory clinical fellow) to confirm the correct diagnosis.

Demographic, clinical, laboratory, and outcome data were collected from the ICU and hospital pathology databases. These databases were used to identify the patients with a diagnosis of cancer together with the case notes review. The diagnosis of cancer, the presence of a malignant tumour, had been previously made by the attending clinicians as per the hospital’s routine diagnostic pathways and was confirmed by histopathology in 16 out of 19 cases. Data on age, gender, and comorbidities such as history of chronic lung disease, heart disease, chronic kidney disease, chronic liver disease, diabetes mellitus and human immunodeficiency virus (HIV) infection were collected. Clinical data included severity of disease scores SAPS (Simplified Acute Physiology Score) II, APACHE (Acute Physiology and Chronic Health Evaluation) II and SOFA (Sequential Organ Failure Assessment) as well as the oxygenation index (ratio of partial pressure arterial oxygen and fraction of inspired oxygen [PaO_2_/FiO_2_ or P/F ratio]) at the time of ICU admission. Additional data included the use of vasoactive drugs (inotropes and vasopressors), main clinical signs and symptoms (heart rate, temperature and blood pressure). The SAPS II, APACHE II, and SOFA scores were calculated as previously described [5–9]. The need for organ support systems (mechanical ventilation [invasive and non-invasive] and renal replacement therapy) during the ICU stay was also collected.

Analytical measurements included full blood count (only haemoglobin [Hb] white blood cell [WBC] and platelets reported), C-reactive protein (CRP), sodium, renal function (urea and creatinine) and liver function tests (aspartate aminotransferase [AST] and alanine aminotransferase [ALT]), as well as the coagulation parameters prothrombin time (PT), activated partial thromboplastin time (APTT) and the international normalised ratio (INR).

Microbiological results were obtained from routine sputum and blood cultures, nasopharyngeal swabs for virus by polymerase chain reaction, urinary antigen for pneumococcal and *Legionella* infections and serology for *Mycoplasma* and other atypical organisms as requested by the on-duty clinicians.

### 2.3 Outcome measurements

Mortality and length of stay in hospital and ICU, respectively, were considered as outcomes.

### 2.4 Statistical analysis

Data were analysed using SPSS version 17 software (USA). For comparison between categorical variables (gender, requirement for mechanical ventilation, inotropes or vasopressors, renal replacement therapy, ICU and hospital mortality), Chi-square or Fisher’s exact test were used as appropriate and the Mann-Whitney test for the comparison of two continuous variables (age, days between hospital and ICU admission, severity scores [APACHE II, SAPS II and SOFA], duration of mechanical ventilation, ICU and hospital length of stay, highest heart rate, highest temperature, lowest systolic and diastolic blood pressure, lowest PaO_2_/FiO_2_ ratio, levels of Hb, WBC, CRP, platelets, INR, PT, APTT, sodium, urea, creatinine, AST and ALT). All reported *p*-values are two-tailed, and are considered statistically significant when *p* < 0.05. All data are represented as median (interquartile range [IQR]) unless otherwise stated.

## 3. Results

Of the 3,669 patient admissions to our ICU during the study period, 309 had a primary coding diagnosis of pneumonia. Of these, 96 patients were found to meet our inclusion criteria for the diagnosis of CAP and were included in the analysis.

### 3.1 Characteristics of patients

Nineteen (19.8%) patients with CAP had an existing diagnosis of cancer at the time of admission to ICU. These included 12 (63.2%) patients with haematological malignancy and 7 (36.8%) with solid organ cancer (including metastatic breast cancer, metastatic testicular cancer, lung cancer, pancreas cancer and renal cancer). Of the 19 cancer patients, 8 were in complete remission at the time of the pneumonia episode. The cause of CAP was identified in a minority of patients (29 [30.2%]). In the patients with cancer an organism was only identified in 4 patients, and included adenovirus *(n* = 2), cytomegalovirus *(n* = 2) and *Mycoplasma pneumoniae* (*n* = 1). For patients without cancer, the identified organisms included *Streptococcus pneumoniae* (*n* = 7), *M. pneumoniae* (*n* = 6), *Staphylococcus aureus* (*n* = 4), influenza A virus *(n* = 3), parainfluenza virus *(n* = 2), *Moraxella catarrhalis* (*n* = 1), *Pseudomonas aeruginosa* (*n* = 1) and *Coxiella burnetii* (*n* = 1).

Demographics, co-morbidities, severity of the disease, need of organ support and use of vasoactive drugs by the study population according to the presence of cancer are summarised in Table 1. There were no statistically significant differences in age, gender or co-morbidities between those with and those without cancer. Patients with a diagnosis of cancer had a longer median time interval between hospital and ICU admission than those without cancer (1 vs 2 days, *p* = 0.049). On admission to ICU, patients with cancer had significantly higher APACHE II (median 25 vs 20; *p* = 0.009), SAPS II (median 51 vs 43; *p* = 0.039) and SOFA (median 12 vs 9; *p* = 0.018) scores. During ICU admission, there were no statistically significant differences in the proportion of patients with or without cancer receiving mechanical ventilation or renal support or in the duration of mechanical ventilation. However, a significantly greater proportion of patients with cancer received vasopressors (89.5% vs 63.6%, *p* = 0.030). On admission to ICU, patients with cancer had significantly lower diastolic blood pressure (median 40 vs 50 mmHg; *p* = 0.026), haemoglobin levels (median 8.6 vs 10.0 g/dl; *p* = 0.010) and higher sodium levels (median 142 vs 139 mmol/l); *p* = 0.020) but no significant differences in WBC, platelets, CRP, clotting (PT, APTT and INR), renal function (urea and creatinine) or liver function (AST and ALT) (Table 2). There were no significant differences in heart rate, temperature, systolic blood pressure or oxygenation index.

**Table 1:** Characteristics of the community-acquired pneumonia population (*n* = 96), according to the presence of cancer

Characteristic	Cancer				*p*-value
	No (*n* = 77)		Yes (*n* = 19)		
	Median (IQR)	Patients *n* (%)	Median (IQR)	Patients *n* (%)	
Age (years)	60 (43–72)		60 (39–72)		0.956
Gender (male)		45 (58.4)		11 (57.9)	1.000
Co-morbidities					
Chronic lung disease		19 (24.7)		1 (5.3)	0.110
Heart disease		21 (27.3)		2 (10.5)	0.147
Chronic kidney disease		10 (13.0)		4 (21.1)	0.467
Chronic liver disease		8 (10.4)		0 (0.0)	0.350
Diabetes mellitus		10 (12.0)		1 (5.3)	0.687
HIV infection		6 (7.8)		0 (0.0)	0.595
Days before ICU admission	1 (0–3)		2 (1–5)		0.049
Severity on admission to ICU					
APACHE II	20 (14–24)		25 (20–29)		0.009
SAPS II	43 (34–53)		51 (42–62)		0.039
SOFA	9 (4–12)		12 (10–13)		0.018
Organ support during ICU admission					
Duration of MV (days)	5 (1–15)		4 (2–25)		0.530
MV		65 (84.4)		17 (89.5)	0.729
Invasive		58 (75.3)		16 (84.2)	0.708
Non-invasive		7 (9.1)		1 (5.3)	
Renal replacement therapy		24 (31.2)		7 (36.8)	0.785
Inotropes/vasopressors during ICU admission		49 (63.6)		17 (89.5)	0.030

ICU, intensive care unit; MV, mechanical ventilation; HIV, human immunodeficiency virus; IQR, interquartile range

**Table 2:** Univariate analysis of physiological and blood test parameters of the community-acquired pneumonia population on intensive care unit admission (*n* = 96), according to the presence of cancer

Characteristic	Cancer		*p*-value
	No (*n* = 77)	Yes (*n* = 19)	
	Median (IQR)	Median (IQR)	
Physical finding			
Highest heart rate (bpm)	111 (95–127)	115 (109–142)	0.214
Highest temperature (°C)	38 (37–38)	38 (37–38)	0.590
Lowest SBP (mmHg)	90 (80–101)	90 (81–95)	0.655
Lowest DBP (mmHg)	50 (40–60)	40 (35–50)	0.026
Lowest PaO_2_/FiO_2_ (mmHg)	117 (82–171)	102 (78–163)	0.580
Laboratory values			
Haemoglobin (g/dl)	10.0 (8.6–2.3)	8.6 (7.7–9.9)	0.010
White blood cells (×10^9^/l)	11.08 (7.45–13.68)	10.76 (5.65–18.70)	0.956
CRP(mg/l)	115 (40–235)	114 (76–247)	0.822
Platelets (×10^9^/l)	192 (120–287)	150 (113–324)	0.459
INR	1.4 (1.2–1.6)	1.4 (1.3–1.8)	0.380
PT (s)	17.6 (16.3–21.1)	18.3 (16.3–23.8)	0.397
APTT (s)	36.5 (31.6–45.8)	38.7 (31.0–46.4)	0.981
Sodium (mmol/l)	139 (135–143)	142 (139–145)	0.020
Urea (mmol/l)	10.4 (4.7–15.2)	9.9 (6.1–18.0)	0.594
Creatinine (µmol/l)	88 (65–193)	128 (51–280)	0.584
AST (IU/l)	42 (24–97)	33 (29–98)	0.858
ALT (IU/l)	31 (16–51)	31 (16–69)	0.887

bpm, beats per minute; SBP, systolic blood pressure; DBP, diastolic blood pressure; PaO_2_/FiO_2_, ratio of partial pressure arterial oxygen and fraction of inspired oxygen; CRP, C-reactive protein; INR, international normalised ratio; PT, prothrombin time; APTT, activated partial thromboplastin time; AST, aminotransferase; ALT, alanine aminotransferase; IQR, interquartile range

### 3.2 Outcomes

There were no significant differences in the length of stay in ICU or hospital between patients with or without cancer (Table 3). Patients with cancer had a markedly increased ICU (68.4% vs 31.2%, *p* = 0.004) and hospital (78.9% vs 33.8%, *p* = 0.001) mortality. There were no significant differences in mortality between patients with active cancer and those in remission (63.6% vs 75.0%, p = 1.000).

**Table 3:** Length of stay and mortality in patients with community-acquired pneumonia (*n* = 96) in intensive care unit (ICU)/hospital, according to the presence of cancer

Characteristic	Cancer				*p*-value
	No (*n* = 77)		Yes (*n* = 19)		
	Median (IQR)	Patients *n* (%)	Median (IQR)	Patients *n* (%)	
Length of ICU stay (days)	8 (3–17)		8 (3–38)		0.712
Length of hospital stay (days)	17 (11–35)		21 (7–47)		0.963
ICU mortality		24 (31.2)		13 (68.4)	0.004
Hospital mortality		26 (33.8)		15 (78.9)	0.001

IQR, interquartile range

## 4. Discussion

Similar to previous data [3], our study demonstrates that patients with cancer and CAP have a considerably increased mortality compared to patients without a co-existent diagnosis of cancer. However, this could reflect reduced use of ICU for patients with cancer and CAP rather than differences in disease severity. By restricting our study to patients admitted to ICU, we have shown that an existing diagnosis of cancer was associated with a markedly increased risk of poor outcome for patients with CAP even when there have been no restrictions on suitability for intensive care support. Our cohort mortality was more than double in the patients with cancer even though 8 were in remission at the time of the pneumonia and despite an identical median age to patients without cancer.

There are several plausible reasons why patients with cancer and CAP may have worse outcomes than patients without cancer. Immune dysfunction, previous antibiotic therapy and prior hospitalisations may increase the risk of infection with resistant or more difficult pathogens in cancer patients. However, in our cohort pneumonia due to resistant or difficult to treat organisms was rare. Recently, it has been shown that patients with cancer that develop acute respiratory distress syndrome (ARDS) have lower survival rates (55.2% vs 24.3%) [10]. However, in this study patients with cancer did not have significantly worse severity of lung injury (with similar P/F ratios), and the requirement for and duration of mechanical ventilation did not differ to patients without cancer. Instead, in our cohort, the poor survival of cancer patients with CAP was associated with an increased illness severity at the time of ICU admission with 5, 8 and 3 point differences in median APACHE II, SAPS II and SOFA scores, respectively. In addition, there was an increased requirement for vasopressors and a lower diastolic blood pressure on admission in the patients with cancer, suggesting that this group had an increased incidence of septic shock. Although the survival of cancer patients with septic shock has previously been reported to not be too dissimilar from mixed populations with septic shock [11], an increased prevalence of septic shock would be expected to translate into increased mortality. Our findings are similar to published data on non-CAP critically ill patients with cancer that also show an increased incidence of septic shock [12–14].

A recent study of cancer patients with acute respiratory failure has demonstrated that the time between the onset of respiratory symptoms and admission to intensive care of more than 2 days was independently associated with increased mortality [15]. In our cohort, CAP patients with cancer were admitted to the ICU on average a day later following hospital admission compared to those without cancer (IQR 1–5 vs 0–3 days). These data suggest one potential reason for the increased illness severity and septic shock at the time of admission to ICU for cancer patients with CAP could be delayed transfer to ICU, either due to failure to recognise illness severity or perhaps because of a reluctance to involve ICU in patients with known cancer. Improved recognition of severe sepsis and an early decision for intensive care management may potentially improve the outcome of this patient group.

The limitations of this study are its relatively small sample size and those associated with the retrospective study design. Due to the small proportion of patients with cancer, analyses comparing the outcomes of those with solid organ and haematological malignancy could not be performed and statistical adjustment for confounding factors was not carried out. Our results indicate the need for a larger, preferably multicentre prospective study to confirm our findings and clarify the reasons for the higher mortality of CAP in patients with a diagnosis of cancer. Furthermore, the identification of cases relied on coding and information in the case notes (information bias). It reflects the real-world scenario and therefore the selection bias is inherent to the on-duty clinicians that made the decision to admit the respective patients with or without cancer.

In conclusion, compared to non-cancer patients, cancer patients with CAP have a lower diastolic blood pressure, and increased illness severity scores and requirement for vasopressor support at the time of ICU admission, and a higher risk of death. These results suggest the increased mortality of CAP in patients with cancer could be partially due to a delay in recognising the need for intensive care support in cancer patients compared to non-cancer patients.
